# Long COVID and Lung Involvement: A One-Year Longitudinal, Real-Life Study

**DOI:** 10.3390/medicina61020304

**Published:** 2025-02-10

**Authors:** Motoc Nicoleta Stefania, Claudia Toma, Cosmina Ioana Bondor, Ruta Victoriua Maria, Petrariu Florin, Man Milena Adina

**Affiliations:** 1 Department of Medical Sciences—Pulmonology, Faculty of Medicine, “Iuliu Hatieganu” University of Medicine and Pharmacy, 400012 Cluj-Napoca, Romaniamanmilena50@yahoo.com (M.M.A.); 2 Department of Pulmonology, “Carol Davila” University of Medicine and Pharmacy, 020021 Bucuresti, Romania; 3 Department of Medical Informatics and Biostatistics, Faculty of Medicine, “Iuliu Haţieganu” University of Medicine and Pharmacy, Louis Pas-teur Str., no. 6, 400349 Cluj-Napoca, Romania; cosmina_ioana@yahoo.com; 4 “Leon Daniello”, Clinical Hospital of Pulmonology, 400371 Cluj Napoca, Romania; 5 Department of Environmental Health and Hygiene, “Grigore T. Popa” University of Medicine and Pharmacy, 700946 Iași, Romania; florin.petrariu@umfiasi.ro

**Keywords:** long COVID syndrome, post-COVID-19, post COVID interstitial lung disease

## Abstract

*Background and Objectives*: Long COVID as a condition typically manifests itself three months after the initial onset of SARS-CoV-2 infection, with symptoms persisting for a minimum of two months. The aim of the present research was to identify potential predictors of post-COVID-19 syndrome (long COVID-19) and to evaluate factors associated with the presence of post-COVID-19 interstitial lung disease and functional decline. *Materials and Methods:* 210 patients hospitalized for confirmed SARS-CoV-2 infections mild, moderate, severe, and critical form have been evaluated at 3, 6 and twelve months. *Results:* Among them only one has been with a suspicion of interstitial lung disease after one year, the rest had no change in the lung. No risk factor from the baseline/3-month or 6-month evaluations significantly influenced patients’ status at 12 months. The longer the duration of hospitalization, the lower the FVC and DLCO were at 3 months, but the longer the duration of hospitalization, the higher the number of symptoms at 3 months and 6 months. In a multivariate linear regression analysis, the number of hospitalization days remained statistically significant only for the number of symptoms at 3 months and 6 months. *Conclusions*: Long COVID seems to be related to the severity of the initial disease, and among the most persistent. Post-COVID-19 interstitial lung disease does not seem to be a frequent entity, as in our study only 0.5% out of 210 patients had it.

## 1. Introduction

Coronavirus disease 2019 (COVID-19) is caused by the severe acute respiratory syndrome coronavirus [1,2]. In March 2020, the World Health Organization officially declared it a pandemic [1,3]. When it comes to the severity of COVID-19, most cases present as mild nonspecific symptoms with no lung involvement. In contrast, moderate cases exhibit more severe symptoms and are characterized by lung involvement (less than 50%), as seen on computerized tomography (CT) scans [4]. Roughly 5% to 8% of all infected individuals present with a severe form of COVID-19, experiencing hypoxia and bilateral lung infiltrates, and may require mechanical ventilatory support [5]. In some cases, a subset of individuals with COVID-19 may continue to experience symptoms for an extended period, and in certain instances these may persist for at least six months. This condition is often referred to as “long COVID”, “long-haul COVID”, or “post-COVID syndrome” [6]. According to World Health Organization (WHO), long COVID is a condition typically manifesting itself three months after the initial onset of COVID-19, with symptoms persisting for a minimum of two months [7]. The incidence of long COVID is estimated to be around 10–30% among non-hospitalized cases, 50–70% among hospitalized cases, and approximately 10–12% among vaccinated individuals. Long COVID encompasses a range of newly developed conditions including respiratory issues, cardiovascular problems, thrombotic and cerebrovascular diseases, type 2 diabetes, encephalomyelitis/chronic fatigue syndrome, and dysautonomia, notably postural orthostatic tachycardia syndrome (POTS) [8]. Various biomarkers have been proposed for the diagnosis of long COVID-19. Aside from clinical manifestations, systemic inflammatory markers like D-dimer, C-reactive protein (CRP), interleukin-6 (IL-6), procalcitonin, and neutrophil count, as well as pulmonary function tests (FVC, DLCO) or findings from high-resolution computed tomography (HRCT), have been included [9]. More than one third of severe COVID-19 survivors exhibited fibrotic-like changes (such as traction bronchiectasis, parenchymal bands, and honeycombing) six months after illness onset, while two thirds of participants showed either complete radiographic resolution or residual ground-glass opacities (GGO) with interstitial thickening. A 12-month follow-up CT scan in a subset of patients who had fibrotic interstitial lung abnormalities (ILAs) at the six-month mark revealed that over two thirds had stable fibrotic ILAs, with a slight improvement in the others. Although the etiology of COVID-19 and subsequent pulmonary fibrosis is not yet fully understood, previous coronavirus pandemics showed that acute respiratory distress syndrome (ARDS) can cause lasting pulmonary fibrosis and significantly affect quality of life [10,11]. Risk factors for ARDS-related interstitial lung abnormalities are represented by old age, acute illness severity, and the duration of mechanical ventilation [12]. In COVID-19 patients, factors such as being aged over 50 years old, having ARDS, a higher baseline CT lung involvement score, smoking, hypertension, lower SaO2, and secondary bacterial infections were identified as predictors of fibrotic-like changes in the lungs [12,13]. Existing evidence suggests that pre-existing chronic interstitial lung disease may be more susceptible to SARS-CoV-2 infection and their general prognosis is worse [14]. Local protective pulmonary mechanisms have reduced the incidence of ARDS-related interstitial lung disease and the severity of respiratory involvement, and apparently the remaining lung damage diminishes within the initial 12 months [15]. There is growing concern regarding the potential development of interstitial lung disease (ILD), as fibrotic changes have been observed as early as three weeks post-infection. These fibrotic changes had a peripheral distribution, consistent with the predominant distribution of ground glass opacities and consolidation observed during the acute illness. The continued effects of post-COVID ILD (PCILD) and the urgent need for new targeted treatments pose a major challenge, particularly when factoring in the significant indirect economic consequences of the pandemic [16].

The aims of the study were to assess risks factors for long COVID-19 syndrome and post-COVID-19 interstitial lung disease. As the primary outcome we sought to identify potential predictors of post-COVID-19 syndrome (long COVID-19). The second outcome was to evaluate factors associated with the presence of post-COVID-19 interstitial lung disease and functional decline.

## 2. Materials and Methods

The study design: This was a prospective cohort study carried out from November 2021 to November 2022 in a tertiary teaching hospital from Romania.

Study population: Data from 210 consecutively hospitalized patients with confirmed SARS-CoV-2 infections mild, moderate, severe and critical forms of COVID-19 were collected. Initial evaluation (T0) was made in the first day of hospitalization and afterwards at 3 months (T1), 6 months (T2) and 1 year for long COVID-19 (T3). We collected clinical data, imaging data (high resolution computer tomography-HRCT) and pulmonary function testing (PFT): spirometry and carbon monoxide transfer factor (TLCO). All patients were called for a follow at 3 months. The following visits (6 months and 12 months) were required only for the patients that had symptoms, modified lung function testing or imaging. Monitoring persistent symptoms was performed through interviews conducted in person for each patient at 3 months after diagnosis, at 6 months, and at 12 months from. Individual patient information was collected by a center made questionnaire and included demographic aspects (age, gender), smoking status, body mass index (BMI), comorbidities, COVID confirmations, severity of acute infection, initial HRCT severity score, number of the hospitalization days, treatment. At three months, we assessed the number of persistent symptoms, HRCT images, severity HRCT score, PFT, DLCO predicted value for all patients. The interviews were conducted by the study team. Irrespective of the severity of the disease, our hospital’s radiology department conducted high-resolution computer tomography (HRCT) scans of the chest for all patients. Subsequently, these images were reviewed by a team of a radiologist and a pulmonologist throughout the entire study. Common imaging findings in COVID-19 patients encompassed ground-glass opacities (GGO), predominantly with a peripheral and subpleural distribution, often affecting the lower lobes of the lungs and reticulations. In our study, we employed the total severity score (TSS), as proposed by Li et al. [14], to quantify the severity on CT scans. This score comprises five severity grades: 0%, 1–25%, 26–50%, 51–75%, and 75–100% involvement for each of the five lung lobes. A higher TSS indicated a more extensive and severe lung involvement. Notably, we also made separate notations for cases where consolidation appeared on thoracic CT scans in COVID-19 patients, as it might be indicative of bacterial infection [17]. Residual radiographic findings or lung function abnormalities required additional investigation every 3 months, until 1 year after the acute COVID-19. Concerning the lung function testing, initially they were not performed as the patients had COVID-19, and afterwards, they were performed at every 3 months. That is why we don’t have spirometry and TLCO in all patients. Pulmonary function testing, both spirometry and TLCO were performed by the same technician according to the existing international guidelines of good clinical practice [18]. Their interpretation was made according to the ERS/ATS guideline by the same doctor [19]. All patients have signed written informed consent, and the study was approved by the Ethical Hospital Committee had ethical approval no 5/05.2021. COVID-19 severity was defined as per following criteria [20,21]: Mild Disease: Characterized by mild symptoms and the absence of dyspnea or pneumonia; moderate Disease: Indicates the presence of lower respiratory issues as determined through clinical evaluation or imaging, along with a blood oxygen saturation (SaO_2_) level of 94 percent or higher when breathing room air at sea level; Severe Disease: Defined by tachypnea (respiratory rate exceeding 30 breaths per minute), hypoxia (oxygen saturation at or below 93% while breathing room air or a PaO_2_/FiO_2_ ratio of less than 300 mmHg), or more than 50% lung involvement observed in imaging. And critical Care: Involves respiratory failure, shock, or multiorgan dysfunction.

Statistical analysis: Descriptive statistics analyses were conducted to explore the patient’s characteristics at baseline, 3 months, 6 months, and 12 months. Analyses were carried out using the SPSS 25 software. Baseline characteristics of the disease were compared between patients who had symptoms at 3 months with those who did not. All the symptoms present at 3 months were analyzed in univariate analysis followed by multivariate analysis. Based on the type of baseline characteristics and the size of the samples, the Student *t* test for independent samples in the case of numerical characteristics, normally distributed and/or large samples, the Mann–Whitney non-parametric test in the case of non-normally distributed and small samples, the Chi-square test in the case of qualitative characteristics with a high number of expected frequencies, or the Fisher exact test in the case of lower expected frequencies were used. For the qualitative characteristics, which were statistically significant, relative risk (RR) and 95% confidence intervals were computed. The linear relationship between two numerical characteristics without extreme cases was determined by Pearson’s r coefficient of correlation, and the non-linear relationship was computed with a rank method (Spearman’s r). In the same way, the symptoms at 6 and 12 months were analyzed based on the same initial characteristics, but also based on the characteristics at 3 months and 6 months, respectively. The multivariate analysis methods were chosen according to the type of dependent characteristics. For dichotomial characteristics, logistic regression was chosen. Each symptom at 3/6 months was examined in a multivariate logistic regression and entered into the model as the dependent variables, where the independent variables were those significant ones in the univariate analysis. Each model was controlled for age and gender. Only for those variables which were significant were the multivariate odds ratio (OR) and 95% confidence interval of the OR reported. For numerical characteristics, linear regression was chosen. Each numerical parameter of the disease at 3/6 months was examined in a multivariate linear regression, and entered into the model as the dependent variables, where the independent variables were significant in the univariate analysis. Each model was controlled for age. Only for those variables which were significant were the coefficient B and 95% confidence interval of the coefficient reported. There were not enough data to conduct a multivariate analysis at 12 months. A *p* value < 0.05 (two-tailed) was statistically significant.

## 3. Results

A total of 210 patients with confirmed SARS-CoV-2 infection were evaluated. A total of 19 (9.0%) out of them required intensive care unit hospitalization. Patients’ initial characteristics are detailed in Table 1. Out of 210 patients treated for SARS-CoV-2 infection, 191 required evaluation at 3 months, 57 patients at 3 months, and only 15 patients at 12 months (see Table 2). At baseline, 100% of the patients had dyspnea and fatigue, and about half had cough, exertion dyspnea, or other symptoms. After 3 months, from 191 evaluated patients, only about 57% were symptomatic and at 12 months only 13 (see Table 3).

The most important factor in symptoms’ persistence was the duration of hospitalization.

Table 2 portrays the characteristics of the evaluated patients from hospitalization (T0) to the subsequent follow-ups at 3 months (T1), 6 months (T2), and 12 months (T3). We checked if the patients had symptoms or not (cough, dyspnea, fatigue, exertion dyspnea, or any other symptoms that could relate to the SARS-CoV-2 infection), we checked changes in the CT or abnormalities (such as ground glass, reticulation, bronchiectasis), or anything that was abnormal, and lung function FVC and TLCO.

At 6 months’ evaluation: The most frequent symptoms related to COVID-19 at 6 months’ evaluation were nonspecific pain syndrome, fatigue, and some psychiatric symptoms. They all seem to be related to older age, COVID-19 severity, days of hospitalization, and the place they were taken care of (pulmonology wards versus intensive care unit) (see Table 3):

As long as the COVID syndrome definition referred to the persistence of symptoms at 3 months, we tried to identify symptoms, lung function abnormalities, or any changes on the CT that could explain why these patients still had symptoms at 6 months. We did not find any risk factor from the baseline, 3 months’, or 6 months’ evaluation that significantly influenced patients’ status at 12 months. The duration of hospitalization was associated with persistent symptoms at 3 and 6 months, and lower lung function values at 3 months (FVC and TLCO) (Table 3). In the multivariate linear regression analysis, the number of hospitalization days remained statistically significant only for the number of symptoms at 3 months and 6 months (Table 3). Only 1 out of the initial 210 patients remained with CT changes after 12 months’ follow up.

## 4. Conclusions

In the present research we sought to identify potential predictors of post-COVID-19 syndrome (long COVID-19). As a second outcome we tried to evaluate factors associated with the presence of long post-COVID-19 interstitial lung involvement. Out of 210 patients hospitalized for confirmed mild, moderate, severe, or critical infection, only 57 had symptoms at 6 months and only 15 patients at 12 months. No risk factor from the baseline, 3 months’, or 6 months’ evaluations significantly influenced patients’ status at 12 months. The duration of hospitalization was associated with persistent symptoms at 3 and 6 months, and lower lung function values at 3 months (FVC and TLCO). In the multivariate linear regression analysis, the number of hospitalization days remained statistically significant only for the number of symptoms at 3 months and 6 months. COVID-19 is classified as a systemic disease because it can have detrimental effects on various organs, including the cardiovascular, gastrointestinal, nervous, hematopoietic, and respiratory systems. However, it is primarily recognized as a respiratory condition [2,22]. Due to globalization, an infectious disease can rapidly spread from one continent to another within hours. This underscores the significance of international coordination in responding to new outbreaks of infectious diseases [13]. The COVID-19 pandemic presented significant challenges for the Romanian healthcare system [22]. Despite notable progress in the development and utilization of COVID-19 vaccines, the pandemic caused by the SARS-CoV-2 coronavirus still exerts pressure on healthcare systems worldwide [23]. Long COVID affects individuals of all ages and is linked to various severities of the acute phase of the disease. The highest percentage of long COVID-19 diagnoses occurs between the ages of 36 and 50 years. Surprisingly, most long COVID-19 cases are found in non-hospitalized patients who have had mild acute illness. This is significant because non-hospitalized patients with mild symptoms make up most of all COVID-19 cases [9]. While most COVID-19 symptoms are typically mild for around 80% of outpatients, certain studies have indicated that 70–80% of patients experience one or more persistent symptoms [24,25]. In our study, although the symptoms were still present at 6 months, at 12 months only a few patients still had symptoms (13 out of the evaluated 15). This might be due to the inhomogeneous COVID-19 patient selection; we had not only severe patients that stayed longer but also mild patients that had fewer days of hospitalization, so this could explain the small number of symptomatic patients. We considered, nonetheless, that the selection of patients with various forms of disease is more realistic and could give us a more realistic prevalence of the long COVID syndrome. The World Health Organization (WHO) estimated that up to 20% of COVID-19 patients experience lingering symptoms for months following infection, and they recognized that this condition is clearly a matter of public health concern. In some cases, these symptoms can persist for years [9]. National data from the UK have suggested that up to 36% of individuals with long COVID experience shortness of breath to some extent, and 26% of them develop signs and symptoms of lung impairment [10]. The above-mentioned symptoms were also present in our study in relatively small percentages. Data suggest the existence of a “hidden pandemic” of long-term complications in individuals who have had severe COVID, with these issues resolving within 3 months of hospitalization in 50% of the patients [20,26,27]. Researchers in Italy have reported that 60 days after the onset of the disease, 87.1% of discharged COVID-19 patients still experience at least one symptom, and 55% experience three or more symptoms. These symptoms often include dyspnea (shortness of breath), chest pain, fatigue, and a reduced quality of life. Despite the establishment of case definitions, there are currently no widely accepted clinical diagnostic criteria for “long COVID”. However, as of 1 October 2021, there is a new International Classification of Diseases, Tenth Revision, Clinical Modification (ICD-10) code for unspecified post-COVID conditions, which is U09.9. This code has been approved by the CDC (Centers for Disease Control and Prevention) [28]. Four potential domains have been identified, which include post-intensive care syndrome, post-viral fatigue syndrome, long-term COVID-19 syndrome, and permanent organ damage [29]. Different authors have used several terms to describe prolonged symptoms following COVID-19 illness, such as “Long COVID-19”, “post-acute COVID-19”, “persistent COVID-19 symptoms”, “chronic COVID-19”, “post-COVID-19 manifestations”, “long-term COVID-19 effects”, “post-COVID-19 syndrome”, “ongoing COVID-19”, “long-term sequelae”, or “long-haulers” as synonyms. Most recently, the term “post-acute sequelae of SARS-CoV-2 infection” (PASC), “long-COVID-19”, and “post-acute COVID-19” have been utilized. Symptoms, signs, or abnormal clinical parameters that persist for two or more weeks after the onset of COVID-19 and do not return to a healthy baseline can potentially be considered long-term effects of the disease [30]. The most common effects were fatigue (58%), headache (44%), attention disorder (27%), hair loss (25%), and dyspnea (24%) [4]. The recent guidelines from the National Institute for Health and Care Excellence (NICE) provide a clear and comprehensive definition of the terminology used to characterize this condition. The term “long COVID” encompasses both the persisting symptomatic phase of COVID-19 (lasting 4–12 weeks) and the post-COVID-19 syndrome (extending beyond 12 weeks) [8,31]. However, it is essential to conduct further research to determine the clinical significance and long-term implications of the remaining radiographic observations and lung function abnormalities [32]. This condition is especially significant within vulnerable populations [33]. Respiratory symptoms, such as dyspnea, continued to be prevalent for weeks to months following the initial diagnosis of COVID-19. In some cases, these symptoms were associated with a decrease in diffusion capacity, which directly correlated with the persistence of ground-glass opacities observed in high-resolution computed tomography (HRCT) [27]. Persistent chest imaging abnormalities and histopathological findings of lung fibrosis were also found in a majority of survivors of the SARS-CoV-1 2003 outbreak, suggesting that the SARS viruses may lead to a more severe fibroproliferative response compared to other types of pneumonia. Pulmonary fibrosis can occur as a complication of respiratory infections, with radiographic abnormalities suggestive of fibrosis persisting in about 20% of non-mechanically ventilated individuals and around 72% of mechanically ventilated individuals even months after hospitalization [32]. Post-COVID fibrosis has been described as organizing pneumonia, interstitial lung disease, or fibrotic lung disease, but a more suitable term is “fibrotic-like” lesions [33]. In our research, only one patient remained with lung abnormalities at 1 year. Most patients presented with lung abnormalities at 3 months and six months. The existing literature [13,14,15,16,17] shows that the interstitial lung abnormality termed post-COVID interstitial lung disease (PC-ILD) is a sequela in patients with severe COVID as well as ARDS, i.e., critical patients. We had only 19 critically ill patients so this might be the main reason why in our study the post-COVID-19 interstitial lung involvement was almost non-existent. The other reason could be the fact that, in general, ARDS associated with interstitial lung disease is non-progressive. Therefore, in a small sample of patients such as our sample, PC-ILD might be non-existent. During a 6-month follow-up, around half of the patients showed at least one abnormality in their chest CT scans [27,33]. In a recent meta-analysis, the prevalence of post-COVID fibrosis was found to be 44.9%, which is lower than that observed during the previous SARS epidemic (62%) but higher than the prevalence in MERS cases (33%) [23]. Fibrosis was clinically confirmed in 56% of patients who had moderate COVID symptoms and in 71% of patients with severe symptoms, three months after recovering from COVID. About 6% of patients continue to require supplemental oxygen at 60-day follow-up. The reported risk of developing fibrosis after recovering from a SARS infection is around 20% [34]. The development of fibrotic-like abnormalities is independently associated with factors such as the initial severity of illness, a CT score exceeding 18, an extended hospital stay, admission to the ICU, prolonged mechanical ventilation, a history of smoking, chronic alcoholism, elevated levels of CRP and IL-6 during the acute phase of the illness, and shorter blood leukocyte telomere length [31,32]. Multiple studies indicate that the likelihood of developing long COVID rises with advancing age. In the Hadler study, patients with fibrotic complications had an average age of 59 years, whereas non-fibrotic patients were notably younger, with an average age of 48.5 years. The elderly population, particularly those with severe COVID-19 symptoms or underlying health conditions, faces the greatest risk of developing post-COVID fibrosis [34]. The frequency of PCPF was 44.9%, and there was no notable difference between genders (53.8% were male, and 46.2% were female) [33]. As reported by Vasarmidi [15] and Rai [16], the incidence of COVID-induced fibrosis could surpass 30% [34]. These radiographic abnormalities are associated with declines in lung function, cough, and frailty [23,33]. Patients who have recuperated from moderate-to-severe COVID are estimated to have a 2–6% risk of developing fibrosis. The prevalence of COVID-induced fibrosis falls somewhere between 10 and 15 cases per 10,000 individuals in the general population, which is 10–100 times higher than the risk of idiopathic lung fibrosis. SARS and COVID exhibit similar pathogenetic features, follow analogous patterns in resolving sequelae during the first 12 months after diagnosis, and share comparable rates of respiratory pathologies (23.7% for SARS and 33% for COVID), as well as persistent fibrotic lung damage (27.8% for SARS and 38% for COVID) over the years [34]. Apart from the symptoms, individuals with long COVID are described as having a diminished quality of life, work-related challenges, effects on physical and cognitive capabilities, reduced health-related quality of life, and limitations in societal engagement. They may necessitate comprehensive care that includes support from social services [33]. Different medications have been experimented with [34]. In our study, although the patients had symptoms and CT changes at 3 months, most of them disappeared at 6 months and only 15 patients out of 210 required evaluation at 12 months. This might be due to the inhomogeneity of patient selection. As we have mentioned in previous studies [14,15], in Romania all patients were hospitalized regardless of the disease severity and lung involvement, and post-COVID-19 seemed to be more frequent among those with severe forms of COVID-19. On the initial CT we found some evidence of fibrotic changes (see Table 1) that we found only in one patient at 12 months. We did not have previous CT for this patient, or the patient’s number was too small. The most common symptoms were as described in the literature, namely dyspnea and fatigue, and were associated in the end with disease severity as appreciated through the number of hospitalization days and CT involvement. Although we consider that our study sheds light on some aspects of long COVID, we must acknowledge the limitations: it is a single-center study, even if it is a large hospital, with a small number of participants, with a heterogenous population, it is not a clinical trial, we do not have an initial evaluation of lung function (maybe the patients had some previously undiagnosed respiratory disorders), and there were not enough patients at 12 months to accurately appreciate lung involvement.

Conclusion: long COVID syndrome seems to be frequent among patients with SARS-CoV-2 infection, regardless of its severity. The most common manifestations appear to be non-specific pain syndrome, fatigue, psychiatric symptoms, and a decrease in exercise tolerance. The longer the duration of hospitalization the higher the number of symptoms at 3 and 6 months. After one year of follow-up, only 1 out of the 210 initially recruited patients remained with lung involvement on the CT. Our patients had various forms of COVID-19 severity, which might explain the few cases of post-COVID interstitial lung disease as, usually, it is described among patients with severe forms of infection. So we might conclude that, in a general cohort of COVID-19 patients, lung involvement after one year is insignificant and most COVID-19-related symptoms are resolved after 6 months. We do consider that the descriptions of long-term sequelae of SARS-CoV-2 infection are still to be presented.

## Figures and Tables

**Table 1:** Patients’ clinical characteristics at the initial evaluation.

Parameters	Baseline Patients = 210
Male gender, no. (%)	111 (52.9)
Age (years), median (25th–75th percentile)	62 (53; 68)
Pack a year index	20 (10; 35)
Active smoker, no. (%)	48 (22.9)
Ex-smoker, no. (%)	57 (27.1)
Never smoker, no. (%)	105 (50)
Comorbidities	176 (83.8)
BMI (kg/m^2^), median (25th–75th percentile)	29.98 (26.88; 34.02)
Cardiovascular disease, no. (%)	65 (31)
Respiratory disease, no. (%)	32 (15.2)
Diabetes, no. (%)	35 (16.7)
Arterial hypertension, no. (%)	106 (50.5)
Gastric disease, no. (%)	53 (25.2)
SARS-CoV-2 infection type, no. (%)	
Mild	40 (19)
Moderate	40 (19)
Severe	116 (55.3)
Critical	2 (1)
No data	12 (5.7)

no.—number; BMI—body mass index.

**Table 2:** Patients’ characteristics at initial evaluation (T0), at 3 months’ (T1), 6 months’ (T2), and 12 months’ (T3) follow up.

	T0	T1	T2	T3
Patients evaluated, no.	210 (100.0%)	191 (91.1%)	57 (27.1%)	15 (7.1%)
Symptoms, no. (%)	210 (100.0)	181 (86.2)	57 (100.0)	13 (6.2)
Cough, no. (%)	97 (46.2)	57 (27.1)	12 (5.7)	5 (2.4)
Dyspnea, no. (%)	210 (100.0)	125 (59.5)	44 (21.0)	9 (4.3)
Fatigue, no. (%)	210 (100.0)	129 (61.4)	34 (16.2)	6 (2.9)
Exertion dyspnea, no. (%)	100 (47.6)	7 (3.3)	26 (12.4)	8 (3.8)
Other symptoms, no. (%)	110 (52.4)	27 (12.9)	11 (5.2)	4 (1.9)
CT SEVERITY * (TSS—total severity score), no. (%)				
Normal	9 (4.3)	1 (0.5)	N/A	N/A
Mild	16 (7.6)	125 (59.5)	N/A	N/A
Median	23 (11)	34 (16.2)	N/A	N/A
Severe	64 (30.5)	18 (8.6)	N/A	N/A
Interstitial lung abnormalities no. (%)	20 (9.5)	80 (38.1)	11 (5.2)	1 (0.5)
Corticoterapy, no. (%)	110 (52.4)	40 (19)	N/A	N/A
Oxygentherapy, no. (%)	100 (47.6)	8 (3.8)	N/A	N/A
Other treatments, no. (%)	N/A	83 (39.5)	N/A	N/A
FVC %, arithmetic mean ± st. dev.	N/A	98.06 ± 19.87	99.3 ± 16.87	92.15 ± 14.79
DLCO %, arithmetic mean ± st. dev.	N/a	78.57 ± 22.17	79.95 ± 20.62	71 (68; 82)

* initial CT 111 patients, CT at 3 months for 178 patients, CT at 6 months 47 patients, CT at 12 months for 10 patients; N/A—the patients did not performed a chest CT or required any kind of treatment.

**Table 3:** Baseline characteristics that have significantly influenced the persistence of some parameters that indicate COVID-19 severity at 6 months’ follow up, univariate and multivariate analysis.

Symptoms at 6 Months Evaluations	Baseline Characteristics		Bivariate Analysis				Multivariate Analysis	
			Without Symptom	With Symptom	*p*	OR, 95% CI	OR, 95% CI	*p*
Nonspecific Pain Syndrome	Age		68 (61; 74)	58 (54; 63)	0.006		0.85 (0.74; 0.98)	0.022
	Obesity		3 (6.8)	5 (38.5)	0.011	3.83 (1.67; 8.78)		
	CT severity	Normal	3 (15)	1 (10)	0.009			
		Mild	3 (15)	1 (10)				
		Medium	1 (5)	6 (60)				
		Severe	13 (65)	2 (20)				
Fatigue	Male		18 (78.3)	14 (41.2)	0.006	0.55 (0.35; 0.85)		
Psychiatric Symptoms	ICU Admission		4 (10)	2 (66.7)	0.047	12.33 (1.31; 115.87)		
Decreased exercise tolerance	Corticosteroid therapy at 3 months		10 (37)	13 (72.2)	0.021	2.49 (1.06; 5.82)		

## Data Availability

All data are available upon request. The raw data supporting the conclusions of this article will be made available by the authors, without undue reservation.
